# Impact of Covid-19 on mental health of Portuguese health professionals – longitudinal study, phase I

**DOI:** 10.1192/j.eurpsy.2022.1268

**Published:** 2022-09-01

**Authors:** D. Machado, A. Borges, C. Laureano

**Affiliations:** Leiria Health Center, Psychiatry And Mental Health, Leiria, Portugal

**Keywords:** health professionals, Covid-19, Impact on mental health

## Abstract

**Introduction:**

The Covid-19 pandemic brought enormous challenges for health professionals. As in past epidemics, the uncertainty, danger and fear of contamination and an excessive load of work under dramatic conditions may contribute to aggravate the mental health of health professionals.

**Objectives:**

Explore how the Covid-19 crisis impacted the mental health of healthcare workers and how their mental status relates with perspectives on the recent past and near future.

**Methods:**

A longitudinal study will be applied in two phases, Q1 and Q2, one year apart, to evaluate depression, anxiety and post-traumatic stress among health professionals from a healthcare center in Portugal. Phase Q1 is concluded and comprised the *Depression, Anxiety and Stress Scale* (DASS-21), the *Impact of Event Scale – Revised* (IES-R) and a questionnaire about the past and the future.

**Results:**

The IES-R scale revealed that nurses are at a higher risk of developing post-traumatic stress disorder (PTSD) than other professionals. The levels of depression and anxiety in the DASS-21 show no significant differences. Interestingly, professionals who worked almost exclusively at inpatient wards show higher levels of depression, anxiety and stress than those who worked at several hospital units (emergency, inpatient and outpatient units). A positive correlation was found between depression and anxiety and negative perspectives about the past and the future.

**Conclusions:**

Covid-19 posed a terrible challenge for health professionals. Its impact on the mental health of healthcare workers may be significant even after the pandemic is under control.

**Disclosure:**

No significant relationships.

